# Unraveling Bacillus Calmette-Guérin (BCG) Therapy Side Effects in Bladder Cancer: A Tale of Triumph Over Treatment Challenges

**DOI:** 10.7759/cureus.50498

**Published:** 2023-12-14

**Authors:** Tomas Escobar Gil, Oscar F Borja Montes, Abu Baker Sheikh

**Affiliations:** 1 Internal Medicine, University of New Mexico School of Medicine, Albuquerque, USA

**Keywords:** adverse drug effect, cytotoxic chemotherapy, severe sepsis, sepsis, immunotherapy, bacillus calmette-guérin (bcg), antimicrobial therapy, oncology, bladder cancer

## Abstract

This case report presents a 66-year-old male with a complex medical history, including testicular cancer, chronic obstructive pulmonary disease, obstructive sleep apnea, tobacco use disorder, erectile dysfunction, and obesity. The patient exhibited recurrent gross hematuria, leading to a comprehensive workup. Cystoscopy revealed a bladder tumor, prompting transurethral resection and mitomycin C instillation. Subsequent intravesical Bacillus Calmette-Guérin (BCG) therapy was initiated but resulted in severe sepsis during maintenance. Despite initial suspicion of BCG-induced sepsis, further evaluation suggested a reaction with chemical cystitis. Treatment involved brief antimicrobial therapy, and the patient's condition improved. This case highlights the challenges in managing BCG therapy complications, emphasizing the need for prompt intervention, careful monitoring, and consideration of risk factors. Patient education and vigilant follow-ups are crucial for addressing potential long-term effects.

## Introduction

Bacillus Calmette-Guérin (BCG) therapy stands as a pivotal modality in the treatment arsenal for non-muscle invasive bladder cancer (NMIBC), offering a unique approach through immunotherapy [1]. The treatment involves the intravesical instillation of live attenuated Mycobacterium bovis, derived from the tuberculosis bacillus, with the aim of provoking a robust local immune response within the bladder [2]. It is a standard treatment for high-risk non-muscle invasive bladder cancer [1]. While BCG has demonstrated remarkable efficacy in reducing the recurrence and progression of superficial bladder tumors, its application is not devoid of challenges, including the potential for disseminated BCG infection (which occurs in less than 5% of patients) and infusion reactions [3]. Studies have reported a reduction in recurrence rates, and complete response rates ranging from approximately 30% to 70% for patients with NMIBC receiving BCG therapy after transurethral resection of bladder tumors (TURBTs) [3].

Understanding how BCG works is fundamental to appreciating its therapeutic potential. The live attenuated mycobacteria stimulate a local immune response by activating macrophages and T lymphocytes [4,5]. This immune cascade not only targets and destroys cancer cells within the bladder but also induces a systemic immune response [5]. The mechanism involves the release of various cytokines, such as interferon-gamma and tumor necrosis factor-alpha, orchestrating an immune-mediated assault on cancer cells [4]. BCG therapy, therefore, serves not only as a direct attack on existing tumor cells but also as a mechanism for bolstering the body's natural defenses against bladder cancer [6].

Despite its success, BCG therapy carries inherent risks. Disseminated BCG infection, though rare, remains a serious concern. This occurs when the instilled mycobacteria escape the confines of the bladder, disseminating through the bloodstream and potentially affecting distant organs. It often necessitates treatment with RIPE therapy (rifampin, isoniazid, pyrazinamide, and ethambutol), although there are some concerns about BCG treatment-resistant strains [7]. Concurrently, infusion reactions, including severe sepsis, can manifest as a result of the heightened immune response induced by BCG [3,8,9]. These complications necessitate a delicate balance in the management of patients undergoing BCG therapy.

## Case presentation

A 66-year-old male patient presented with a history of testicular cancer (managed with radical orchiectomy), chronic obstructive pulmonary disease, obstructive sleep apnea, tobacco use disorder, erectile dysfunction, and obesity. The patient presented with gross hematuria persisting for approximately one week, a symptom he had experienced one to two months prior, leading to treatment for a urinary tract infection despite being asymptomatic at the time. Additionally, he described nocturia and a slightly slower urine stream, with the effect of tamsulosin on these symptoms being uncertain. Despite these urinary symptoms, prostate screenings remained consistently unremarkable. Further evaluation revealed a post-void residual test (PVR) result of 8 and an overall symptom score of 3, indicating low burden.

**Figure 1:**
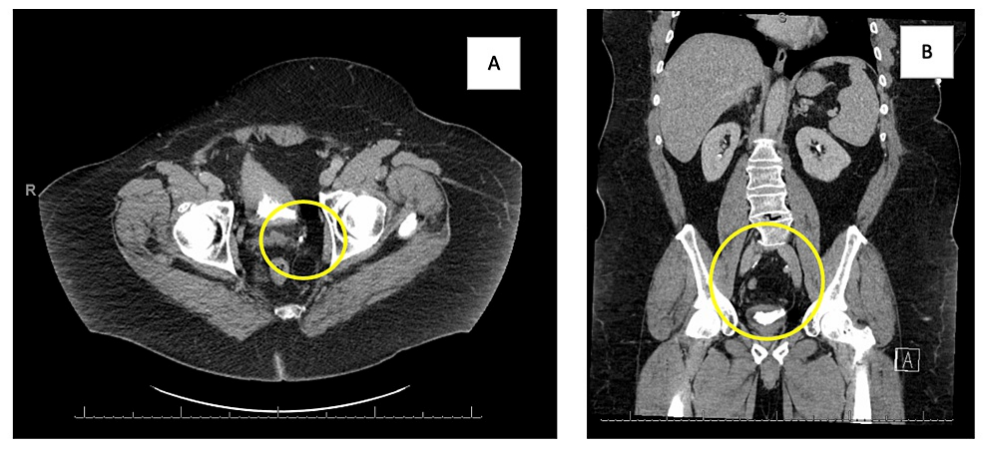
Abdominal CT (A) a filling defect on the left side of the bladder consistent with a transitional cell carcinoma. After surgical resection and mitomycin C treatment, (B) the resolution of the filling defect.

Given the patient's medical history and smoking status, a comprehensive workup for gross hematuria was initiated. Cystoscopy performed by the urology team revealed a bladder filling defect at the left ureteral orifice and a calcified left-wall bladder tumor on a stalk, raising suspicion for transitional cell carcinoma. Consequently, TURBT was performed, followed by the instillation of mitomycin C. After the initial treatment, the patient was scheduled for weekly intravesical BCG therapy for a duration of six weeks. Subsequent follow-up cystoscopies did not detect any recurrence. A CT of the abdomen and pelvis before and after treatment with tumor resection and mitomycin C can be seen in Figure 1A, 1B.

During maintenance BCG therapy, the patient returned to the ER, following a three-week cycle of BCG therapy, presenting with shortness of breath and dysuria, but without hematuria. Upon admission, he met the criteria for severe sepsis, with fever reaching 40.6°C, tachycardia peaking at 110 bpm, respiratory rate at 32 breaths per minute, increased oxygen requirement, and lactic acidosis (2.80). Given an apparent urinary tract infection/sepsis, there was a high suspicion of BCG-induced sepsis and disseminated BCG infection. Upon consultation, the infectious disease team recommended the initiation of RIPE therapy and vancomycin. Acid-fast bacilli, urine, and blood cultures were consistently negative. As the patient's condition improved symptomatically and clinically, the initial clinical impression favored a BCG infusion reaction with chemical cystitis, given the timing of his presentation (on the day of the last instillation). Consequently, the RIPE therapy and antibiotics were discontinued after 48 hours. The patient reported a return to baseline status and has remained under follow-up care since.

## Discussion

**Figure 2:**
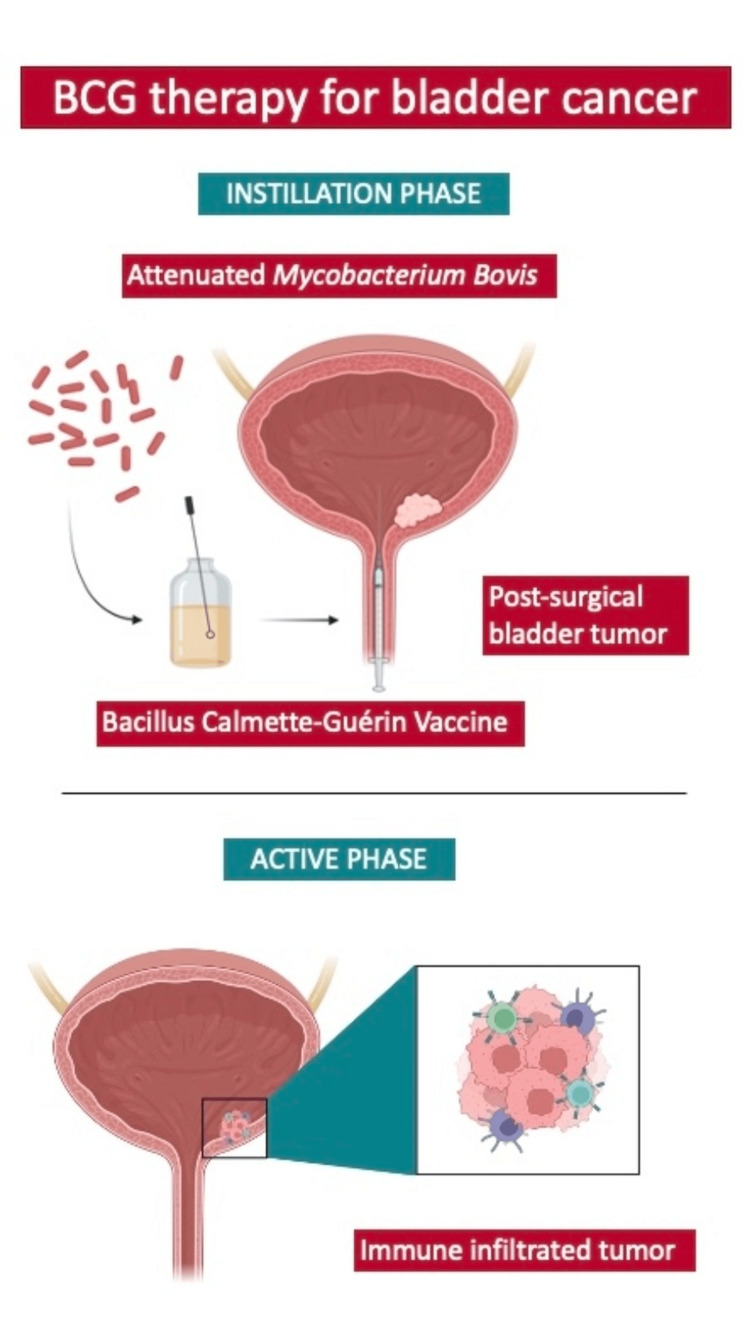
Mechanism of action of Bacillus Calmette-Guérin (BCG) therapy for bladder cancer In the instillation phase, BCG therapy is instilled into the bladder. The active phase comprises the activation of T lymphocytes, which develop tumor-specific killing activity. Figure Credit: Author Tomas Escobar Gil

BCG for bladder cancer is one of the oldest forms of immunotherapy [1]. A diagram depicting its mechanism of action can be seen in Figure 2. While BCG is effective for both immunocompetent and immunocompromised patients, it can lead to systemic side effects in 30%-40% of patients and potentially life-threatening complications, including distant organ infections [10]. Management of these effects necessitates immediate intervention, continuous monitoring, and cautious medication use. A very small fraction of treated patients develop life-threatening sepsis [3,7]. BCG sepsis is treated with antimycobacterial antibiotics (RIPE) and glucocorticoids [3,6]. Known risk factors such as urothelial barrier disruption or concurrent urinary tract infections should be considered before BCG administration to minimize complications [10]. It is crucial to provide patient education and maintain vigilant follow-ups for potential long-term effects.

Alternative approaches to non-muscular invasive bladder cancer are surgical [10]. TURBT is the mainstay therapy for non-muscle invasive disease, with subsequent chemotherapy instillations [10]. Additionally, en bloc bladder tumor removal (EBRT) has been proposed as a preferable technique, reducing cell spillage, and decreasing surgical risks. However, BCG immunotherapy stands as the sole conservative intervention proven to deter progression in high-risk NMIBC [10].

The future of bladder cancer treatment includes immunotherapy with checkpoint inhibition, targeted therapies, and antibody-drug conjugates [2]. Checkpoint inhibitors, which potentiate the body's immune response against cancer cells, are a key focus in ongoing trials for bladder cancer. Targeted therapies, such as FGFR inhibitors, offer a more precise treatment option for patients with specific genetic alterations. Additionally, antibody-drug conjugates like enfortumab-vedotin provide a combination of monoclonal antibodies and chemotherapy, minimizing damage to healthy tissues [1]. The evolving landscape suggests that the integration of these novel therapies with established methods will likely shape the standard of care, offering improved outcomes and more tailored treatment strategies for bladder cancer patients [10].

## Conclusions

In conclusion, BCG therapy remains a cornerstone in the management of NMIBC, exhibiting efficacy in reducing tumor recurrence. However, the presented case underscores the intricate challenges associated with BCG treatment, including chemical cystitis and severe sepsis and the differential diagnosis of the rare but serious complication of disseminated BCG infection. The future of bladder cancer treatment, as discussed, is evolving with the exploration of immunotherapy and targeted therapies, offering potential alternatives or complementary approaches. As we navigate this evolving landscape, the significance of prompt recognition, accurate diagnosis, and vigilant monitoring of BCG-induced side effects becomes paramount. While BCG therapy continues to be a valuable tool in bladder cancer treatment, ongoing research and a personalized approach are essential to optimize its benefits while minimizing potential complications, thus ensuring the best possible outcomes for patients facing this challenging disease.

## References

[1] Lange C, Aaby P, Behr MA, Donald PR, Kaufmann SH, Netea MG, Mandalakas AM (2022). 100 years of Mycobacterium bovis bacille Calmette-Guérin. Lancet Infect Dis.

[2] Lenis AT, Lec PM, Chamie K, Mshs MD (2020). Bladder cancer: a review. JAMA.

[3] Ramalingam S, Gunasekaran K, Arora H, Muruganandam M, Nagaraju S, Padmanabhan P (2021). Disseminated BCG infection after intravesical BCG immunotherapy of bladder cancer. QJM.

[4] Han J, Gu X, Li Y, Wu Q (2020). Mechanisms of BCG in the treatment of bladder cancer-current understanding and the prospect. Biomed Pharmacother.

[5] Biot C, Rentsch CA, Gsponer JR (2012). Preexisting BCG-specific T cells improve intravesical immunotherapy for bladder cancer. Sci Transl Med.

[6] Ward Grados DF, Ahmadi H, Griffith TS, Warlick CA (2022). Immunotherapy for bladder cancer: latest advances and ongoing clinical trials. Immunol Invest.

[7] Watts MR, Taylor PC, Sintchenko V (2011). Implications of isoniazid resistance in Mycobacterium bovis Bacillus Calmette-Guérin used for immunotherapy in bladder cancer. Clin Infect Dis.

[8] Chang SS (2019). Re: disseminated mycobacterium bovis infection post-kidney transplant following remote intravesical BCG therapy for bladder cancer. J Urol.

[9] Takase R, Hagiya H, Fujimori T (2023). Super acute-onset disseminated BCG infection: a case report. J Infect Chemother.

[10] Dobruch J, Oszczudłowski M (2021). Bladder cancer: current challenges and future directions. Medicina (Kaunas).

